# Bordeaux 2018: Wine, Cheese, and γδ T Cells

**DOI:** 10.3389/fimmu.2019.02544

**Published:** 2019-10-25

**Authors:** Karen Edelblum, Kenth Gustafsson, Daniel J. Pennington, Benjamin E. Willcox, Julie C. Ribot

**Affiliations:** ^1^Department of Pathology, Immunology and Laboratory Medicine, Center for Immunity and Inflammation, Rutgers New Jersey Medical School, Newark, NJ, United States; ^2^Infection, Immunity and Inflammation Program, London, United Kingdom; ^3^Molecular and Cellular Immunology Section, UCL Great Ormond Street Institute of Child Health, London, United Kingdom; ^4^Barts and the London School of Medicine, Blizard Institute, Queen Mary University of London, London, United Kingdom; ^5^Cancer Immunology and Immunotherapy Centre, Institute of Immunology and Immunotherapy, University of Birmingham, Birmingham, United Kingdom; ^6^Faculdade de Medicina, Instituto de Medicina Molecular, Universidade de Lisboa, Lisbon, Portugal

**Keywords:** gamma delta T cells, conference report, recent advances, ongoing research, futures perspectives

## Introduction

**Figure 1:**
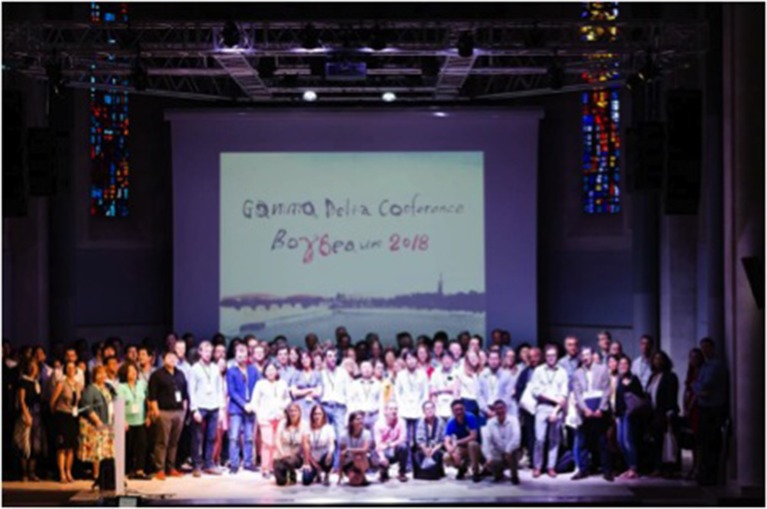
Group picture of the participants during the 8th International Gamma-Delta T Cell Conference in the Agora, Domaine du Haut-Carré, Bordeaux, France.

The 8th “International conference” was held at the University of Bordeaux, 7–10 June 2018. The lead organizer was Julie Déchanet-Merville who together with the other members of the organizing committee; Maria Mamani Matsuda, Matthias Eberl, Myriam Capone, Lionel Couzi, Hannah Kaminski, Layal Massara, Pierre Merville, Sonia Netzer, Angela Pappalardo, Charlotte Domblides, and Vincent Pitard, provided an interesting and exciting programme covering recent progress and developments in all aspects of γδ T cell research. The conference had a very good attendance with 294 delegates from 28 countries on the five continents (Figure 1). In total, 160 abstracts were submitted; resulting in 57 oral presentations and 103 poster presentations. In addition, eight “special lectures” were given after invitation. A “best poster” and “oral presentations” competition was held, covering all sessions, resulting in 10 and 5 awards, respectively (funded by AAI). Thirty-six travel awards were provided for students and post-docs by generous donations from NIH, European Federation of Immunological Societies and Gamma Delta Therapeutics Inc.

The conference presentations were divided into “basic” and “applied/clinical” sessions. Below we provide a snapshot of the progress we mainly heard about from the oral presentations during these 4 days.

## γδ TCR Repertoires and Specificities

Alongside the need for new γδ TCR ligands to be identified, the requirement to align ligand specificities of individual γδ TCRs with properties of the overall γδ TCR repertoire was discussed in this session.

Erin Adams (University of Chicago, USA) highlighted some of the distinct and unconventional features of current structurally defined γδ TCR/ligand interactions (1–3) relative to αβ TCRs, including a dominance of the genetically diverse TCRδ CDR3 region in binding to the T10/T22 ligand.

Sarina Ravens (Prinz laboratory, Hannover Medical School, Germany) presented an analysis of the TRD repertoire in children from Europe or Africa, and highlighted relatively slow changes in diversity over the first years of life, including over time periods where relevant challenges such as CMV infection (4) likely took place, or after measles vaccination. A discussion point was whether these kinetics reflected differential regulation of such responses in early childhood.

Ben Willcox (University of Birmingham, UK) summarized an analysis of the human liver γδ T cell compartment (5) which highlighted high levels of clonal focussing consistent with an effector cell status, and also revealed a functionally specialized subset of hepatic γδ T cells that are liver-resident and clonotypically distinct from those in blood.

Juan Carlos Zúñiga-Pflücker (University of Toronto, Canada) exploited the *in vitro* OP9DL system to generate from haematopoietic stem cells γδ T cells that were specific for the melanoma-associated antigens MART-1 and gp100. Although relevant γδ TCRs exhibited MHC-restricted recognition, a point of discussion was whether such specificities would be induced *in vivo* during melanoma (6).

Martin Davey (Willcox laboratory, University of Birmingham, UK) presented an analysis of human Vδ2^+^ T cells (7) highlighting an unrecognized adaptive subset comprising Vγ9^−^Vδ2^+^ T cells. This population is not phosphoantigen reactive, and instead features a TCR-diverse naive repertoire that becomes greatly focussed after CMV infection, alongside differentiation to effector status, similar to Vδ1^+^ T cells (8).

Alina Fichtner (Herrmann laboratory, University of Wurzburg, Germany) presented results on γδ T cells from Alpaca, the first non-primate species shown to possess phosphoantigen- reactive T cells. While other placental mammals share some elements of the phosphoantigen- sensing pathway, alpacas have Vγ9JγP and Vδ2 gene segments, and a single BTN3 molecule that includes a B30.2 domain capable of interacting with IPP/HMBPP. This system may prove useful to help define the minimal requirements for BTN3-mediated phosphoantigen recognition.

## γδ T Cell Regulation by Butyrophilins

Recently, the butyrophilin (Btnl/BTN) family of genes has been shown to regulate the differentiation and activation of γδ T cells (9, 10); however, the underlying mechanisms by which these molecules are activated and influence TCR signaling remains unclear.

Anna Vyborova from Zsolt Sebestyén's laboratory (Utrecht, The Netherlands) generated Vγ9Vδ2 multimers and showed that a Vγ9Vδ2 TCR induces T cell activation through a multistep activation model. The TCR first exhibits a scanning mode that can be enhanced by pamidronate, followed by a recognition mode dependent on CDR3-mediated affinity and the detection of microclusters of BTN3A1 on the cell membrane through Vγ9Vδ2 TCR.

In support of the inside-out model of butyrophilin signaling in response to phosphoantigen, Lola Boutin from Emmanuel Scotet's laboratory (Nantes, France) showed that mutation of the juxtamembrane domain of BTN3A1 can modulate Vγ9Vδ2 T cell activity. This suggests that additional domains outside of the B30.2 region are critical for molecular interactions. Moreover, the formation of BTN3A1/3 heterodimers depends on the context of antigen activation, and that treatment with statins could abrogate these interactions.

A series of reports from Adrian Hayday's laboratory (London, UK) addressed the role of TCR/butyrophilin signaling in epithelial γδ T cell populations.

First, Duncan Mckenzie showed that the expression of Skint1 in keratinocytes focuses TCR expression to the tips of Vγ5Vδ1 dendrites. Loss of Skint1 expression in response to epithelial stress coincides with the dissociation of T cell-keratinocyte contacts. Although Vγ5Vδ1 TCR signal strength was increased following epithelial stress, the loss of cellular interaction alone was not sufficient to activate the γδ T cells, suggesting that a tonic signal maintains TCR activation and a secondary signal is required for a stress-induced response.

Daisy Melandri reported that the ability of intestinal intraepithelial lymphocytes to respond to Btnl1 and Btnl6 is a property of the Vγ7 chain. Further, the interaction with butyrophilins was mediated by the HV4 region of the TCR, and not the CDRs, suggesting that an innate non-clonal region of the Vγ chain regulates responsiveness to these molecules (11).

Pierre Vantourout showed that similar specificity exists among human colonic γδ IELs in which Vγ4, but not Vγ2 IELs were responsive to BTNL3 and BTNL8. *In silico* modeling showed that Vγ4Vδ1 TCR likely directly interacts with BTNL3, and that this binding interaction is distinct from that observed between TCR and CD1d. Together, these reports presented a model in which innate-like TCR reactivity results in the generation of a tonic signal that is required for IEL tissue residence.

Robin Dart presented clinical applications of these findings showing that co-culture of BTNL3 and BTNL8-expressing HEK293T cells with colonic γδ T cells isolated from healthy patients induced TCR downregulation. However, BTNL-dependent TCR downregulation was severely attenuated in Vγ2/3/4 cells isolated from patients with inflammatory bowel disease (IBD), which could be recapitulated following culture of Vγ2/3/4 from non-IBD controls with pro-inflammatory cytokines. Identification of a BTNL3/8 polymorphism that fails to regulate Vγ2/3/4 cells revealed that patients homozygous for this mutation exhibited a higher incidence of ileocecal disease, suggesting that BTNL-γδ dysregulation may predispose individuals to develop IBD.

## γδ T Cell Activation, Regulation and Function

This third session was introduced by David Vermijlen (Brussels, Belgium) who provided a complete overview about the diversity of γδ T cell repertoire in human, as previously presented by Martin Davey (7) and Sarina Ravens (4). Beyond the periphery, David presented provocative new data from RNA sequencing of fetal vs. post-natal thymic γδ T cells, showing that innate γδ T cells are functionally programmed in the fetal—but not post-natal—thymus.

Dmitry Ushakov from Adrian Hayday's laboratory (London, UK) presented a 3i Immunophenotyping analysis of single cell confocal images of T cells and Langerhans cells in the epidermis. In data gathered from over 3,000 individual mice from >550 knockouts, 24 knockouts showed a γδ T-cell-specific phenotype, 20 of these were specific to the skin, and 8 of those were specific to the epidermis.

Weili Xu from Anis Larbi's laboratory (Singapore) showed that in response to aging and cytomegalovirus history, human Vδ2^+^ T cells are more protected from cellular senescence compared to all other αβ and γδ T cells.

Benjamin Gully from Jamie Rossjohn's group (Melbourne, Australia) identified the N-terminal scavenger receptor cysteine rich (SRCR) domain structure of the cell surface co-receptor on bovine γδ T cells, Workshop cluster-1 (WC-1), which allowed insight into potential antigen binding surfaces.

Mathilde Raverdeau from Lydia Lynch's laboratory (Dublin, Ireland) reported that CD27^−^ and CD27^+^ γδ T cells exhibit fundamental differences in their metabolic profiles at steady state. Whereas, IL-17^+^ γδ T cells primarily generate energy through oxidative phosphorylation, those producing IFNγ^+^ use a glycolytic pathway.

Jonathan Boyson (Burlington, VT) showed that heterogeneous expression of Slam receptors mark functional γδ T cell subsets, with SLAMf1 and SLAMf6 expression being associated with IL-17^+^ and IFNγ^+^ γδ T cell populations, respectively. These Slam receptor profiles were established during thymic development, and global deletion of the SLAM adapter protein (SAP) resulted in a loss of thymic RORγt^+^ γδ T cells thus reducing the SLAMf1 IL-17^+^ γδ T cell population.

Tiago Amado from Bruno Silva-Santos's group (Lisbon, Portugal) reported that microR146a was upregulated in CD27^−^ γδ T cells producing IL-17^+^ and functioned to restrict IFNγ production by targeting Nod1 mRNA (12).

## γδ T Cell Homing In Tissues

Increasing evidence over the last several years has revealed the importance of γδ T cells in promoting tissue homeostasis. Identification of crosstalk between γδ T cells and other cells (epithelial, stromal and myeloid) within the tissue microenvironment has shed light on new functional roles for specific γδ T cell subsets under steady-state conditions and expanded our view of how these cells are primed to respond to local infection.

Providing novel insight into the functional role of γδ IELs in the intestine, Karen Edelblum (Newark, USA) showed that Vγ7 IELs were required for shedding of apoptotic intestinal epithelial cells at the villus tip under pathological conditions such as systemic LPS exposure. Further, intravital microscopy showed that this subset of IELs directly interacts nearly half of shedding enterocytes immediately prior to their expulsion into the lumen.

Multiple reports highlighted novel molecular regulators of Vγ6Vδ1 T cell plasticity across a broad range of tissues. Darshana Kadekar from Vasileios Bekiaris's laboratory (Copenhagen, Denmark) reported the identification of a novel intestine-specific RORγT^+^ Tbet^+^ γδ T cell population. Initially, RORγT^+^ γδ T cells populate the neonatal intestine and then upregulate Tbet expression during the first week of life through a STAT5-dependent mechanism. Within the ileal and colonic lamina propria, these double-positive cells co-produce IL-17, IL-22, and IFN-γ.

Claire McIntyre from Vicky Morrison's laboratory (Glasgow, UK) showed that loss of β2 integrin (CD18) expression resulted in a 10-fold expansion of γδ T cells in the lung, spleen, blood, and uterus under steady-state conditions. This substantial increase in γδ T cell number was specific to IL-17-producing Vγ6Vδ1 T cells due to an absence of CD11a (α_L_β_2_), indicating that β2 integrin expression negatively regulates this subset of γδ T cells.

Lydia Lynch (Dublin, Ireland) showed that PLZF^+^ Vγ6Vδ1 T cells produce both TNF and IL-17A in adipose tissue (13). Crosstalk between these innate PLZF^+^ γδ T cells and adipose stromal cells regulates endogenous IL-33 production to maintain core body temperature. Mice deficient in γδ T cells fail to appropriately thermoregulate in response to cold challenge, which may be due to an inability to induce IL-17-mediated brown fat activation necessary for thermogenesis.

Besides fat, Vγ6Vδ1 T cells were also found in the female reproductive tract. Leticia Monin from Adrian Hayday's laboratory (London, UK) reported that uterine γδ T cell compartment is more abundant in young mice <5 weeks of age. These Vγ6Vδ1 T cells secrete IL-17A, IFNγ- or both cytokines within the uterine stroma. Further, uterine γδ T cells confer protection against Candida infection through the recruitment of neutrophils, which is lost in γδ T-cell-deficient mice.

Vγ6^+^ IL-17-producing T cells were also reported to infiltrate the stromal tissue of the testes by Julie Ribot (Lisbon, Portugal). During puberty, expansion of this γδ17 population was mediated by androgen-driven changes in the gut microbiome and myeloid cell IL-23 and IL-1 expression downstream of TLR4. Intra-testicular infection with *Listeria monocytogenes* was more severe and resulted in increased lethality of γδ T cell- or IL-17-deficient mice, indicating that testicular IL-17 producing γδ T cells are critical for limiting local bacterial infection.

## γδ T Cell Evolution and Development

The session opened with an overview of murine γδ T cell development from Daniel Pennington (London, UK). This introduced a consensus view of the stages of γδ T cell development and focused on the factors that affect thymic commitment to subsequent effector fates (i.e., to become γδ17 or γδIFN cells). The idea that γδ17 cells may not be generated from a common αβ/γδ progenitor in the thymus was discussed, and consistent with peripheral data presented earlier in the day by Mathilde Raverdeau from Lydia Lynch's laboratory (Dublin, Ireland), IL-17-, and IFN-γ- producing γδ T cells were revealed to adopt profoundly different metabolic programs at the very earliest stages of their development.

The session continued with two interesting comparative immunology presentations that emphasized how a sole focus on human and rodent biology may provide a distorted perspective. Breanna Breaux (Texas, USA) first described the TCRγ and TCRδ loci in the Florida manatee, which belongs to the afrotherians, a clade of eutherian mammals that includes elephants. For the gamma locus, initial data suggest there are many multiclusters with repeated Vγ and Jγ segments (i.e., high segmental diversity compared with human and mouse). By contrast, the delta locus has restricted combinatorial diversity as only one Dδ and Jδ segment was identified. Nonetheless the CDR3δ region was of a comparable length to those seen in other species. A VHδ segment, that has been observed in frogs, birds, and monotremes, was also identified (a first in a eutherian species).

In the next presentation, Rob Miller (Albuquerque, USA) continued the comparative biology theme by discussing the fifth TCR chain (TCRμ) in non-eutherian mammals (marsupials and monotremes). TCRμ pairs with TCRγ and contains a second Vμ domain (i.e., a third extracellular domain) similar to the VNAR domain that is observed in cartilaginous fish. “γμ cells” are transcriptionally distinct from both αβ T cells and γδ T cells in the opossum and represent ~10% of the T cells in the spleen. The presence of a TCRμ locus and VHδ regions provides an interesting evolutionary perspective to the origin of the TCR loci.

After the brief journey into comparative immunology, Apostol Apostolov (Lyon, France) returned to the session's general theme of γδ T cell development, describing a CD4 fate mapping approach that identified a CD4^+^ bone marrow precursor that could give rise to various subsets of murine γδ T cells.

Juliette Roels (Ghent, Belgium) from Tom Taghon's laboratory followed this by describing an RNA deep sequencing approach on ten human thymocyte subsets that represent various stages of αβ and γδ T cell development. Interesting observations on human vs. mouse T cell development were highlighted; proliferation was largely conserved yet regulation of preTCR components appeared different. γδ T cell biased genes were enriched in NK and CD8 T cell-associated signatures. Moreover, expression of RORγt, c-Maf, and Sox13 were all evident, despite the relative lack of γδ17 T cell development in human.

The next presentation by Sagar (Freiburg, Germany) returned to murine γδ T cell development, this time using a single-cell RNA sequencing approach. The study identified a TCR signal strength-related propensity to develop as either γδ17 or γδIFN cells in the CD25^+^ progenitor subset. It also identified c-Maf as a key regulator of the γδ17 program with Sox-13/c-Maf/RORγt sequential gene expression. Indeed, c-Maf KO mice lacked γδ17 cells and γδ progenitors in these animals appeared to have an increased signal strength gene profile. Finally, c-Maf KO mice were unsurprisingly protected from γδ17-driven immunopathology.

The c-Maf theme was continued by Maria Ciofani (Durham, USA). Deletion (via IL-7-Cre) of c-Maf in all lymphoid progenitors completely abrogated γδ17 cell development, and expression of genes associated with a γδ17 program (e.g., RORγt and Blk) was lost. Deletion of c-Maf in γδ17 cells (via RORγt-Cre) also demonstrated a requirement for c-Maf to maintain the γδ17 cell program. c-Maf expression levels were inversely proportional to signal strength from various transduced γδTCRs (e.g., KN6) and appears to antagonize Tcf-1 function that is a negative regulator of γδ17 cell development (14).

The session ended with Paola Tieppo (Brussels, Belgium) as we again switched to human γδ T cell development. Notably, human fetal γδ T cells are enriched for Vδ2^+^ cells (unlike post-natal γδ T cell populations) that use invariant, public sequences. The generation of these early invariant γδ T cells appears to be dependent on a specific fetal precursor. Interestingly, the transfer of an unidentified RNA binding protein to post-natal hematopoietic progenitors converts them to a fetal mode in which they increase generation of invariant Vδ2^+^ cells.

## New Concepts in Immunology

The evening session was an entertaining departure from the “conventional” session format. The first speaker Thomas Pradeu (Bordeaux, France) adopted an intentionally philosophical approach to understanding the key concepts that underpin immune responses. He introduced the idea of a “discontinuity” theory in which the immune system has evolved to recognize changes from a “normal” state, mainly reacting to changes rather than status quo presence of antigens. Thomas suggested that this theory had advantages over models that suggested danger as a key initiator of immune responses, as it explained scenarios in which danger had not really been present.

This was followed by an equally provocative talk from Adrian Hayday (London, UK). Adrian used the opportunity to discuss (γδ T cell-driven) tissue immunosurveillance in the context of a “validation” theory. Adrian argued that for conventional αβ T cell responses the sensor (i.e., the TCR) crucially requires a validation signal (e.g., B7-mediated) before cells are activated. γδ T cells lack CD28, so what replaces the B7/CD28 validation axis? Skint1 and the BTNL family of genes are related to B7 and have recently been shown to be key TCR-binding regulators of specific tissue resident γδ T cell subsets. Interestingly, there is now evidence that these B7-like molecules may interact with the TCRγ chain in a non-CDR-mediated fashion. Adrian left the audience to consider whether tissue resident T cells required that sensor input via conventional γδTCR-CDR/ligand interactions must be validated by tissue stress surveillance (i.e., is the tissue “normal”?), also through the TCR, but by this intriguing family of B7-like molecules.

## γδ T Cell Function in Infection and Inflammation

Besides recent studies on the mechanisms required for steady state γδ T cell homing and homeostasis in tissues where they can contribute to local physiology, a series of presentations has focused on their functions in a pathogenic context. While their protective role against infection has been widely recognized in different mouse models and human diseases, γδ T cells can also be associated with deleterious outcomes, by exacerbating the inflammatory response.

The session was introduced by Willi Born, who gave a brilliant overview of the research program he has been leading for the past 30 years, together with his colleague Rebecca O'Brien. He mainly focused on the atypical regulatory role of IL-4 producing Vγ1^+^ γδ T cells (15) and its impact on B cell development, activation and IgE production (16, 17). On behalf on the γδ T cell community, we would like to warmly acknowledge Willi and Rebecca for their important contribution to the field and wish them a great and well-deserved retirement.

Christophe Paget (Tours, France) reported a key protective cross talk in the lung between IL-1β neutrophils and IL-17-producing Vγ6 T cells in a model of invasive pneumococcal infection. This process was mediated by the neutrophilic NLRP3 inflammasome activated by macrophage-derived TNF-α bacterial pneumolysin (18).

Johnny Guo from Paul Thomas' laboratory (Memphis, USA) demonstrated a critical role for IL-17 producing γδ T cells upon neonatal influenza infection that enhanced the production of IL-33 in lung epithelial cells. This important cross talk was shown to promote a protective type 2 response, namely by inducing the secretion of amphiregulin by Tregs and ILC2s. Importantly, this mechanism may also exist in human, as suggested by the positive correlation observed between IL-17 and IL-33 in the nasal fluid of infected children.

Besides IL-17, Murad Mamedov from Mark Davis' laboratory (Stanford, USA) identified Macrophage-Colony-Stimulating-Factor (M-CSF) as a novel protective cytokine produced by an oligoclonal Vγ1Vδ6.3+ γδ T cell subset in a mouse model of malaria infection. Interestingly, γδ T cells expanded rapidly after resolution of acute parasitemia, in contrast to αβ T cells that expanded at the acute stage and then declined. This dynamic was also observed in *Plasmodium falciparum* infected subjects, suggestive of a key mechanism that could be targeted for the prevention of malarial recurrence in humans (19).

By analyzing cord blood samples from neonates of women infected with placental malaria, Cristiana Cairo (Baltimore, USA) showed that phosphoantigens released by *Plasmodium falciparum* during placental sequestration primed fetal Vδ2^+^ γδ T cells, altering their phenotype (increased PD-1 expression) and function (reduced cytotoxic potential).

Further advances in our understanding of the anti-infectious function of γδ T cells have been made by the team of Zheng Chen (Chicago, USA) through studies in a macaque model of tuberculosis (20). In this congress, he presented a vaccination strategy based on phosphoantigen HMBPP-producing attenuated Listeria vector, which targets specifically the Vγ9Vδ2 T cell subset, therefore mounting efficient memory-like responses that reduce pulmonary bacterial burden after challenge.

Allen Cheung (Hong Kong) reported the presence of a novel population of Vδ2^+^ γδ T cells that accumulate in the gut of HIV patients upon acute infection. These cells were characterized by the constitutive expression of Δ42PD1 (a PD-1 isoform) and tissue homing receptors such as CCR9 and CD103. Further *in vivo* experiments in humanized mice showed that Δ42PD1 interacted with TLR4 to promote innate immune activation and intestinal pathogenesis, thus highlighting a novel mechanism of mucosal inflammation.

Anne Hahn from Thomas Winkler's group (Erlanger, Germany) monitored the TCR repertoire of γδ T cells in different tissues along the timecourse of murine Cytomegalovirus (mCMV) infection. Using a Nur77-GFP reporter assay for TCR activation, she screened for γδ T cell clones that recognize mCMV infected target cells. This work may shed light on the nature of specific Ag for γδ T cells by identifying novel TCR ligands in mice, following up on the work in human previously reported by the groups of Ben Willcox and Julie Déchanet-Merville (21, 22).

Hannah Kaminski from Julie Déchanet-Merville's laboratory (Bordeaux, France) described a paradoxical effect of the mTOR pathway on effector T cell functions. While it is used as an immunosuppressive drug in transplantation, mTOR inhibitors (mTORi) were associated with less CMV infections in transplanted patients. She showed that mTORi increases Vδ2- T cell *in vitro* expansion and IFN-γ production, as confirmed by proteomic analysis of purified Vδ2- γδ T cells from mTORi-treated patients. Mechanistically, she suggested that mTORi could inhibit mTORC1 while inducing a negative feedback increase of AKT phosphorylation, leading to phosphorylation of S6, and expression of T bet. This study parallels previous data from the group of David Pauza on the Vδ2^+^ γδ T cell counterpart (23).

Simone Cuff from Matthias Eberl's laboratory (Cardiff, UK) presented a novel diagnosis strategy based on algorithms that rely on the analysis of local immune fingerprints from patients with acute peritonitis. She showed that incorporating the Vγ9Vδ2 T cell response into machine learning models is key to the production of an accurate immune profile of peritonitis and in particular, to immune profiles associated with specific pathogens.

In addition to their relevance against infections, the three following oral communications focused on the role of γδ T cells in the pathogenesis of autoimmune and inflammatory diseases.

Inga Sandrock from Immo Prinz's laboratory (Hannover, Germany) presented a new knock-in mouse line (Tcrd-GFP-DTR Luciferase mice), in which γδ T cells can be conditionally depleted with diphtheria toxin (24). She showed that acute depletion of γδ T cells in these mice results in protection from IMQ-induced psoriasis and from spondyloarthritis-resembling inflammation induced by *in vivo* overexpression of IL-23. This mouse model revealed compensatory mechanisms for IL-17 production normally mediated by αβ T cells and ILC3 in the constitutive γδ T-cell-deficient mice. Of note, ILC3 compensated for IL-17 production 9 weeks after γδ T cell depletion induced upon diphtheria toxin injection.

Julie Jameson (San Marcos, USA) showed that obesity impaired γδ T cell persistence in the gut, by downregulating adhesion molecules and chemokine receptors (CD103, CCR9). The remaining intestinal γδ T cell functions were dysregulated, as obese mice were more susceptible to DSS-induced severe colitis. Importantly, the process was reversible upon a 7-week administration of a diet inducing weight loss.

Following up on his previous investigation (25), Jun Yan (Louisville, USA) reported a critical role of the IL-1-IL-1R signaling in psoriasis pathogenesis (26). IL-1β induces dermal γδ T cell proliferation and IL-17 production via the IL-1R-MyD88-mTOR signaling pathway. IL-1β also stimulated keratinocytes to secrete chemokines such as CCL20, which chemoattract peripheral CCR6^+^ IL-17^+^ γδ T cells. Interestingly, endogenous IL-1β secretion was regulated by the skin microbiota to maintain dermal IL-17^+^ γδ T homeostasis. The transfer of Corynebacterium isolated from human psoriatic skin on naïve mouse skin significantly stimulated IL-1β production, leading to dermal IL-17^+^ γδ T cell expansion and psoriatic lesions.

## γδ T Cell Function in Cancer

In addition to their role in infection and inflammation, γδ T cells are widely recognize to display key anti-tumor activities through their IFN-γ production and potent cytotoxicity. Notwithstanding, recent data have now highlighted unexpected pro-tumoral functions linked to IL-17-producing γδ T cells. Thus, further studies are required for a better understanding of the potential of γδ T cell modulation in cancer immunotherapy.

Sofia Mensurado from Bruno Silva-Santos' group (Lisbon, Portugal) identified suppressive tumor-associated neutrophils that specifically inhibited the proliferation of pro-tumoral IL-17 producing γδ T cells in mouse. By expressing low levels of the antioxidant glutathione, this subset was shown to be particularly sensitive to neutrophils-derived-reactive oxygen species. Interestingly, she suggested that these findings could be applied to human, as Vδ1^+^ γδ T cells, which contain most IL-17 producing γδ T cells found in cancer patients, also displayed low glutathione levels (27).

José Villacorta Hidalgo from Paul Fisch's laboratory (Freiburg, Germany) analyzed the distribution of γδ T cells infiltrating sentinel lymph nodes in human triple negative breast cancer and suggested that high endothelial venules may be critical to regulate γδ cell entry from the blood into the tumor.

Daniela Wesch from Dieter Kabelitz (Kiel, Germany) demonstrated a critical immunosuppressive role for β-galactoside-binding protein galectin-3 in pancreatic ductal adenocarcinoma and ovarian cancer. Galectin 3 is released by tumor cells and interacts with α3β1 integrin expressed by Vδ2^+^ γδ T cells. While it did not impact on cell-cytotoxicity or survival, galectin 3 clearly impaired Vδ2^+^ γδ T cell-proliferation.

Following up previous work on the identification of NK receptors expression by anti-tumoural Vδ1^+^ γδ T cells (28), Elena Bruni from Domenico Marvilio's laboratory (Milan, Italy) showed that the expression of NKp46 was restricted to Vδ1^+^ (but not Vδ2^+^) γδ IELs and associated with an increased production of granzyme B and IFN-γ. NKp46^+^ phenotype is a feature of IL2/IL15-induced human infant thymic γδ T precursors (29) and correlates with significantly lower tumor progression in CRC patients.

Finally, Jean Jacques Fournié (Toulouse, France) presented recent computational methods that perform cell type-specific quantifications from the transcriptomic analysis of tissue samples. Using CIBERSORT, an algorithm that allows the deconvolution of bulk tumor transcriptomes to find tumor infiltrating lymphocytes (TILs), previous studies have identified γδ TILs as the most significant favorable cancer prognostic cell population (30). By implementing machine learning from purified γδ T cell microarray data, Jean Jacques Fournié reported an updated improved version of CIBERSORT that enumerate and characterize Vγ9Vδ2 γδ TILs in 10,000 cancer biopsies from 50 types of hematological and solid malignancies (31). Jean-Jacques also presented new data on γδ T cell single cell RNA sequencing that define a specific γδ T cell signature enabling characterization of these cells in complex tissues RNA sequencing analyses. This new tool will help pave the way for critical findings that will be highly relevant for immunotherapy.

## γδ T Cells in Immunotherapy

γδ T cells are now fully appreciated as being functionally profoundly different from their αβ T cell counterparts. The increased understanding of their basic biology, as evidenced in other sessions in this conference, has meant that increasing numbers of research groups as well as clinical centers are now considering their use in cancer immunotherapy. In addition, the pharmaceutical and biotech industry are now actively entering this new area of immunotherapy. A number of encouraging oral as well as poster reports during this conference highlighted the considerable progress made in this area since the last γδ T cell conference. They all helped to form the strong impression that γδ T cells will become a very important addition to current immunotherapy strategies, at a minimum, and quite possibly a profound improvement at best—some might go as far as to say, a revolution of future immunotherapy strategies directed at (and possibly beyond) cancer.

Marta Barisa (from UCL Institute of Child Health, London, UK) reported on a new generation of Chimeric Antigen Receptor (CAR) constructs designed to suit γδ T cells. Whilst CARs expressed in αβ T cells provide “signal 1” for activation through the use of endodomains for that purpose, this γδ T cell-specific strategy makes use of the ability of the γδ TCR to provide “signal 1” through “stress” recognition of tumor cell targets. The γδ T cell CAR is instead engineered to provide a co-stimulatory “signal 2” following recognition of a molecular target on a cancer cell. This strategy arguably provides two distinct advantages: firstly, the well-known cancer cell ability to down-regulate molecules that are able to provide natural “signal 2” stimulation (such as MICA/B) is replaced by a “therapeutic signal 2,” and secondly, it avoids on-target, off-tumor activation which is a well-recognized problem in current αβ T cell-based CAR treatment protocols.

Trudy Straetemans presented further results from the Jürgen Kuball group (Utrecht, Netherlands) on their strategy to confer the ability of γδ T cells to recognize “stressed” tumor cells on αβ T cells by expressing a defined γδ TCR in addition to their endogenous αβ TCR (TEGs). They selected a particular γ9δ2 TCR (TEG001) for testing of transduced αβ T cells in a 3D model consisting of multiple myeloma cells within the context of a humanized bone marrow stromal niche. The data presented showed that the tumor cells, but not the stromal cells, were specifically targeted in association with cytokine production. The findings demonstrate the potential clinical utility of this strategy.

B-cell malignancies are able to inhibit Vγ9Vδ2 γδ T cell anti-tumor activities. In her presentation, Barbara Castella (Massimo Massaia, Turin, Italy) showed that several functional impairments contribute to this inhibition of Vγ9Vδ2 γδ T cell reactivities. This includes multifaceted immune check-point expression in the tumor microenvironment. By combining PD-1 and TIM-3 blockade the ability of Vγ9Vδ2 γδ T cells to proliferate was restored. This type of work can be predicted to improve the efficacy of γδ T cell therapies against multiple myeloma as well as other malignancies.

Zhinan Yin (Guangzhou, China) presented some intriguing and hopeful results from *in vitro* expansions and clinical trials using allogeneic Vγ9Vδ2 γδ T cells. *Ex vivo* expansions followed by adoptive transfer of autologous Vγ9Vδ2 γδ T cells can be problematic as these are often impaired in various pathologies. Following an improved *ex vivo* expansion protocol, the allogeneic Vγ9Vδ2 γδ T cells were adoptively transferred to 80 breast cancer patients. Preliminary results indicated that this allogeneic cell transfer was safe, cancer progression slowed down, survival increased and immune function was improved in a majority of the patients, highlighting that the use of allogeneic γδ T cells in cancer immunotherapy constitutes a viable and very welcome alternative to autologous therapies.

Anne-Charlotte Le Floch (Daniel Olive, Marseille, France) reported on further use of their agonist antibody against the Vγ9Vδ2 TCR candidate ligand BTN3A (20.1). They have found that its use increases the cytotoxicity of Vγ9Vδ2 T cells against acute myeloid leukemia cells. They showed that the main mechanism appears to be increased degranulation and expression of DNAM-1 by/on Vγ9Vδ2 T cells.

Biagio Di Lorenzo (Bruno Silva-Santos, Lisbon, Portugal) provided new data on their “Delta One T” (DOT) expanded Vδ1^+^ γδ T cells. The DOT cells were shown to be effective in killing human acute myeloid leukemia cells, including against clones of AML which were chemoresistant. These positive results were also translated into efficient cytotoxicity in a xenogeneic *in vivo* mouse model of AML. These results thus provide a very welcome novel means to potentially improve on the otherwise poor outcomes for AML patients.

Craig Morita (Iowa City, USA) showed data on a combination treatment of human prostate tumors in a xenogeneic mouse model by using Vγ9Vδ2 T cells in combination with PD-1 mAb blockade. The combination treatment reduced tumor burden to nearly to zero after 5 weeks—promising another means by which previously disappointing Vγ9Vδ2 T cell treatments of human tumors can be substantially improved.

In another combination treatment approach, Lawrence Lamb (University of Alabama, Birmingham, USA) demonstrated that using Temozolomide (TMZ) to induce upregulation of NKG2D ligands on human glioma tumors, in a xenogeneic glioma tumor mouse model, drastically increased the efficacy of adoptively transferred GMP-grade γδ T cells efficacy. This study suggests that this combination treatment could now be used in human clinical trials.

Noemie Joalland (Emmanuel Scotet group, Nantes, France) presented striking results from a xenogeneic human/mouse model showing that by combining existing chemotherapeutic and surgical strategies with immunotherapeutic transfer of allogeneic Vγ9Vδ2 γδ T cells and GMP grade aminobiphosphonates can significantly improve the survival of epithelial ovarian carcinoma-carrying animals. The results showed that chemotherapy treatment may not harm the beneficial effects of adoptively transferred γδ T cells.

Hans-Heinrich Oberg (Daniela Wesch, Kiel, Germany) showed data on the use of a tribody directed against the HER2 antigen on cancers of epidermal origin and the CD16 antigen on γδ T cells and NK cells [(HER2)xCD16]. The results from clinical trials in breast cancer, ovarian tumor, and pancreatic cancer patients showed better results than the use of mAb trastuzumab and the main reason for the promising results was shown to be an increased degranulation of immune cells, presumably γδ T cells and NK cells.

## Concluding Remarks

Bruno Silva-Santos (Lisbon, Portugal) closed the congress leaving us with the meeting's main highlights and future perspectives for γδ T cell research. Within the most significant advances made since the previous γδ T cell conference, the growing evidence that some γδ T cell subsets adopt an adaptive biology and clonally expand in response to pathogen infection was highlighted (4, 5, 7, 8). Also within such advances, Bruno mentioned butyrophilins as molecular mediators of tissue surveillance, namely their role in sensing stress, as well as new determinants of effector γδ T cell differentiation including new transcription factors (14) and the contribution from metabolic pathways. Bruno also underlined novel roles of γδ T cells impacting on tissue physiology at steady state, namely regulating thermogenesis (13) and neuroplasticity (32).

The development of γδ T cell based-therapies are part of the exciting future directions that were mentioned. Besides efforts being pursued against infection and autoimmunity, new clinical trials in cancer immunotherapy will be launched in the coming years. We look forward to hearing about the next discoveries in the field: the 9th γδ T cell conference is scheduled for 2020 in Beijing (China).

## Author Contributions

KE and JR wrote the manuscript with the help of KG, DP, and BW.

### Conflict of Interest

The authors declare that the research was conducted in the absence of any commercial or financial relationships that could be construed as a potential conflict of interest.
